# Latent Toxoplasmosis among Breast Cancer Patients in Jahrom, South of Iran

**DOI:** 10.1155/2023/4792260

**Published:** 2023-07-22

**Authors:** Marzeieh Haghbin, Salar Maani, Mohammad Aref Bagherzadeh, Ahmadreza Bazmjoo, Heshmatollah Shakeri, Ali Taghipour, Shahab Falahi, Azra Kenarkoohi, Milad Badri, Amir Abdoli

**Affiliations:** ^1^Research Center for Noncommunicable Diseases, Jahrom University of Medical Sciences, Jahrom, Iran; ^2^Department of Surgery, Jahrom University of Medical Sciences, Jahrom, Iran; ^3^Student Research Committee, Jahrom University of Medical Sciences, Jahrom, Iran; ^4^Zoonoses Research Center, Jahrom University of Medical Sciences, Jahrom, Iran; ^5^Department of Infectious Diseases, Jahrom University of Medical Sciences, Jahrom, Iran; ^6^Department of Parasitology and Mycology, Jahrom University of Medical Sciences, Jahrom, Iran; ^7^Zoonotic Diseases Research Center, Ilam University of Medical Sciences, Ilam, Iran; ^8^Department of Microbiology, Faculty of Medicine, Ilam University of Medical Sciences, Ilam, Iran; ^9^Medical Microbiology Research Center, Qazvin University of Medical Sciences, Qazvin, Iran; ^10^Metabolic Diseases Research Center, Research Institute for Prevention of Non-Communicable Diseases, Qazvin University of Medical Sciences, Qazvin, Iran

## Abstract

**Objective:**

Reactivation of latent toxoplasmosis is the main cause of severe infection among immunocompromised patients, including patients with cancer. Hence, this study is aimed at screening the status of *Toxoplasma gondii* infection among breast cancer patients by serological and molecular methods and determining their associated risk factors in Jahrom County, Fars Province, south of Iran.

**Methods:**

One hundred and seven women with breast cancer (aged 34 to 80 years) were screened for anti-*T. gondii* antibodies (IgG and IgM) during 2019–2020. A questionnaire regarding demographic factors was filled out by participants. Molecular detection was performed by polymerase chain reaction (PCR) using the primer pair targeting the repetitive element (RE) gene of *T. gondii*. The risk factors and demographic data were analyzed by SPSS software (ver. 20, Chicago, IL, USA) using the Chi-squared test.

**Results:**

Anti-*T. gondii* IgG was detected in 45.8% (49/107) of the patients, which indicates latent infection, but anti-*T. gondii* IgM and PCR were negative in all samples. Demographic factors revealed a statistically significant increased *T. gondii* seropositivity among nonmenopause cancer patients (*P* < 0.0005), patients without previous breast cancer (*P* = 0.0001), and human epidermal growth factor receptor 2- (*HER2-*) negative patients (*P* = 0.00002). As such, patients with a history of previous abortions and who were at stages II, III, and IIII of cancer had higher seropositivity rates than patients without a history of previous abortions or who were at stage I cancer, but the statistical analysis was not significant. We did not find a statistically significant association between *T. gondii* seropositivity and other risk factors of toxoplasmosis (e.g., education level, type of water source, washing raw fruits and vegetables, consumption of raw or undercooked meat, and contact with soil, cats, and domestic animal).

**Conclusion:**

A high seroprevalence rate of latent *T. gondii* infection was detected among patients with breast cancer; hence, these patients may be at high risk for reactivation of latent infection. Screening of *T. gondii* infection is recommended to detect active infection among patients with malignancies.

## 1. Introduction

Breast cancer is the most prevalent cancer in women worldwide, with an estimated 2.3 million new cases in 2020 [1]. *Toxoplasma gondii* is an intracellular parasite with a worldwide prevalence among warm-blooded animals and humans [2]. Humans are mainly infected through ingestion of water or food, which is contaminated with oocysts shed by cats or by eating raw or undercooked meat containing *T. gondii* tissue cysts [2]. Congenital transmission occurs in women who acquire their primary infection during gestation and can result in abortion or severe damage to the fetus [2]. In healthy individuals, the infection is usually self-limiting and asymptomatic [3]. However, toxoplasmosis in immunocompromised patients (e.g., cancer patients, organ transplant recipients, and HIV/AIDS-positive individuals) could be a life-threatening infection with fatal outcome [4–8]. Furthermore, reactivation of latent infection may cause severe infection in immunocompromised patients, which results in encephalitis or disseminated infection [2, 8, 9]. In this regard, reactivations of latent toxoplasmosis have been reported in patients with different types of cancer [8–11].

A number of studies revealed a higher occurrence of toxoplasmosis in patients with cancer compared to healthy individuals [7, 12–15]. However, little is known about the prevalence and risk factors of toxoplasmosis in breast cancer patients. Hence, this study is aimed at evaluating the serological and molecular status of *T. gondii* infection among breast cancer patients and determining their associated risk factors in Jahrom, Fars Province, south of Iran.

## 2. Materials and Methods

### 2.1. Study Population

The present study was performed among 107 women (aged 34 to 80 years) who were diagnosed with breast cancer during 2019–2020. All patients were from a referral center in Jahrom County (Khatam-al Anbiya Center of Breast Cancer in Peymanieh Hospital, Jahrom, Iran). This study protocol was approved by the Ethical Committee of Jahrom University of Medical Sciences (IR.JUMS.REC.1398.002). Before sampling, all voluntary participants or their legal representatives gave both verbal and written informed consent. A questionnaire regarding demographic factors was filled out by participants. Furthermore, information such as type of treatment, menopause status, history of previous breast cancer, and human epidermal growth factor receptor 2 (*HER2*) status were obtained from the patient's medical records. The stages of cancer were determined according to the tumor node metastasis (TNM) classification. All patients did not have any immunodeficiency disease. About five milliliters of venous blood samples were collected from each patient; after centrifugation, the serum samples were separated for serologic evaluation, and the buffy coat samples were used for DNA extraction and molecular detection. All methods were carried out in accordance with relevant guidelines and regulations in the declaration (Ethical Approval and Consent).

### 2.2. Serological Technique

The anti-*T. gondii* IgG and IgM antibodies were detected by the enzyme-linked immunosorbent assay (ELISA), with a commercial ELISA kit (Pishtaz Teb, Tehran, Iran) according to the manufacturer's protocol. The diagnostic criteria of IgG and IgM antibodies were defined by cut-off values, and the upper and lower limits of 11 IU/mL were considered as positive and negative, respectively.

### 2.3. Molecular Detection

The buffy coat samples were used for DNA extraction using the phenol–chloroform–isoamyl alcohol method, as described in a previous study [16]. PCR was performed using the repetitive element (*RE*) gene amplifying a region of 529 base pairs (bp) fragments. The primers are highly sensitive and specific for *T. gondii* due to 200–300 replications in the *T. gondii* genome [17]. The PCR primers and cycling conditions were described in previous reports [18, 19]. For each reaction, a positive control (DNA extracted from the RH strain of *T. gondii*) and a negative control (double-distilled water) were included. PCR products were electrophoresed in 2% agarose gel, stained with safe stain (Sinaclon, Iran), and visualized under a UV transilluminator.

### 2.4. Statistical Analysis

The demographic data and risk factors were analyzed by SPSS (ver. 20, Chicago, IL, USA) using the Chi-squared test.

## 3. Results

**Figure 1:**
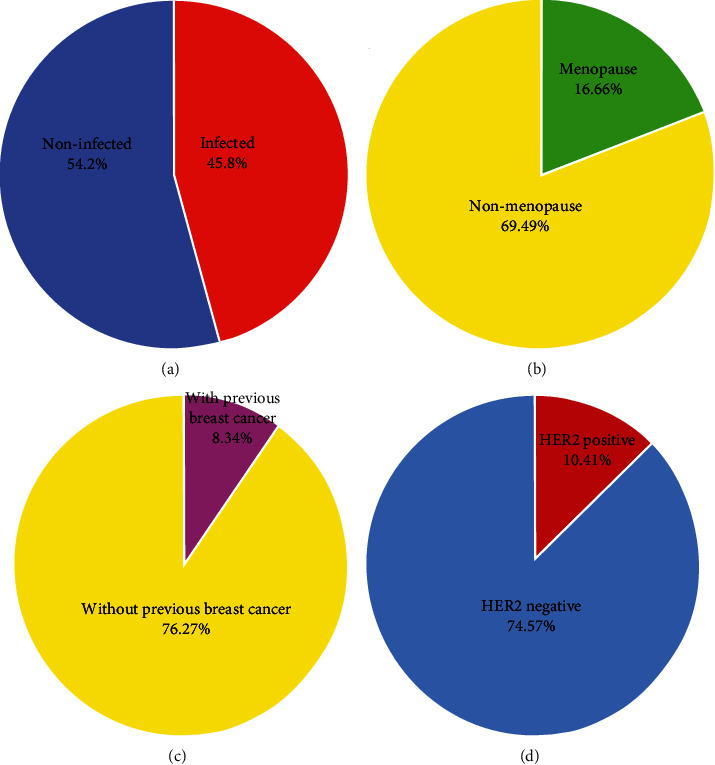
(a) The overall seroprevalence rate of *T. gondii* among breast cancer patients; (b) seroprevalence among menopause and nonmenopause patients (*P* = 0.0005); (c) seroprevalence among patients with and without previous breast cancer history (*P* = 0.0001); (d) seroprevalence among HER2-positive and -negative patients (*P* = 0.00002).

The mean ages of the patients were 55.66 years (±11.66 standard deviation (SD)), ranging from 34 to 80 years old (Table 1). Anti-*T. gondii* IgG was detected in 45.8% (49/107) of the patients, but anti-*T. gondii* IgM was negative in all samples (Figure 1(a)). Furthermore, PCR was negative in all IgG-positive and -negative cases. Demographic factors revealed a statistically significant association between *T. gondii* IgG seropositivity and menopause status, while *T. gondii* seropositivity was 16.6% and 69.49% among menopause and nonmenopause patients, respectively (*P* = 0.0005, Figure 1(b)). *T. gondii* seropositivity rate was 76.27% and 8.33% among patients without previous breast cancer compared to patients with previous breast cancer (*P* = 0.0001, Figure 1(c)). As such, *T. gondii* seroprevalence rate was significantly higher among human epidermal growth factor receptor 2- (*HER2-*) negative than *HER2*-positive patients (74.57% vs. 10.41%, respectively, *P* = 0.00002, Figure 1(d)). A nonsignificant increase *T. gondii* seroprevalence rate was detected among patients who received hormone therapy (74.07%) and radiotherapy (66.66%) than patients who had surgery (26.66%) (*P* = 0.08) (Table 1). Patients who were at stages II, III, and IIII of cancer had a higher seropositivity rates than patients who were at stage I cancer (stage I: 25%, stage II: 53.65%, stage III: 72.97%, and stage IV: 71.42%), but it was statistically not significant (*P* = 0.51) (Table 1). Patients with a history of previous abortion had an increased seroprevalence rate of *T. gondii* than patients without history of previous abortion (66.66% vs. 37.66%, respectively), but the statistical analysis was not significant (*P* = 0.11).

There was not a statistically significant association between IgG seropositivity and other risk factors of toxoplasmosis (e.g., education level, consumption of raw or undercooked meat, washing raw fruits and vegetables, type of water source, and contact with soil, cats, and domestic animal) (Table 1).

## 4. Discussion

Patients with cancer are susceptible to infection with a diversity of pathogens, including *T. gondii* [4, 8, 9, 15]. The result of a meta-analysis indicates that cancer patients have a three times higher risk of toxoplasmosis than healthy individuals (OR = 3.1, 95% CI: 2.5–3.8, *P* < 0.0001) [12]. Toxoplasmosis is typically asymptomatic in immunocompetent individuals, but reactivation of latent infection in immunocompromised patients can lead to a severe infection with a life-threatening outcome [3, 4]. In our study, we performed seromolecular tests to screen for *T. gondii* infection in patients with breast cancer. Overall, anti-*T. gondii* IgG antibody was detected in 45.8% of the patients, but anti-*T. gondii* IgM and PCR were negative in all samples. We use the *RE* gene, which is highly sensitive and specific for *T. gondii* due to 200–300 replications in the *T. gondii* genome [17]. Previous studies regarding the seroprevalence of toxoplasmosis in breast cancer in the north of Iran revealed that 86.4% and 7.6% had anti-*T. gondii* IgG and IgM antibodies, respectively [20]. Kalantari et al. detected *T. gondii* DNA in 10.3% (3/29) of breast and lymph node biopsies in Iran. Additionally, all patients were positive for *T. gondii* IgG and were IgM negative [21].

Other studies were performed in toxoplasmosis and different types of cancer in Iran [12]. Accordingly, in our previous study, among different types of cancer [15], the overall IgG seroprevalence of *T. gondii* was 41.51%, while the seroprevalence among women with breast cancer was 44.44%. Moreover, *T. gondii* IgM was negative in all patients, but PCR was positive in a patient with a high IgG antibody titer. Arefkhah et al. [22] detected anti-*T. gondii* IgG and IgM among 13% and 2.1% of cancer patients in the southwest of Iran, respectively. In the north of Iran. Hosseini et al. [23] detected anti-*T. gondii* IgG and IgM as well as *T. gondii* DNA among 75.4%, 2.57%, and 5.43% of cancer patients, respectively. Saki et al. [24] detected *T. gondii* IgG and IgM among 41.7% and 6.4% of children with cancer in the south of Iran, respectively.

Previous studies in the same region (Jahrom County) revealed that the prevalence rates of toxoplasmosis were lower than in our study. In this regard, Maani et al. [25] assessed the seroprevalence rates of toxoplasmosis among 370 pregnant women in Jahrom City, and accordingly, anti-*T. gondii* IgG and IgM were detected in 29.5% and 0.5% of pregnant women, respectively. Another study in the same region was conducted among 120 pregnant women with a history of recurrent abortion (case group) and 50 women with normal delivery (control group) [26]. The study indicated that anti-*T. gondii* IgG was positive among 17.5% and 14% of the case and control groups, respectively [26]. Although the seroprevalence rates of *T. gondii* in these two studies [25, 26] were lower than in the current study, the comparison of our results with them could not be accurate because the age ranges of our study were between 34 and 80 years old, but the age ranges in these two studies [25, 26] were under 40 years old. It should be noted that the seroprevalence rate of toxoplasmosis could increase with increasing age [27]. Other studies in Fars Province revealed a seroprevalence rate ranging between 7.5% and 29% [25, 28–31] (Table 2).

Moreover, we found a nonsignificantly higher rate of *T. gondii* IgG antibody among patients who had a history of abortion compared with those without a previous history of abortion (66.66% vs. 37.66%) (Table 1). We also found that the rate of abortion was 75% in the first trimester of pregnancy and decreased to 62.5% and 50% in the second and third trimesters of pregnancy, respectively (Table 1). These results are in consistent with the time of abortion in congenital toxoplasmosis, which the risk of abortion due to congenital toxoplasmosis decreases with developing pregnancy [2]. It is well documented that congenital toxoplasmosis is associated with abortion and stillbirth [2, 32–38], but little is known about the history of abortion among cancer patients with toxoplasmosis. It is generally believed that women who have *T. gondii* IgG before pregnancy are immunized against congenital toxoplasmosis [2]. However, our recent reports revealed that *T. gondii* IgG-seropositive women also had a risk of spontaneous abortion [35]. On the other hand, some epidemiologic studies suggest an association between abortion and breast cancer risk, but the results are inconsistent. In this regard, Michels et al. gathered data on 3,958 breast cancer cases and 11,538 controls from seven countries and found a weak association between spontaneous abortion and breast cancer risk [39]. A negative association between spontaneous abortion and breast cancer risk was reported in Greece [40]. A large study in Iran revealed no association between history of spontaneous abortion and breast cancer risk [41], although a meta-analysis demonstrated that induced abortion might increase the risk of breast cancer only in parous women [42].

We found an increased in *T. gondii* seropositivity rate among patients who received hormone therapy, radiotherapy, and patients who were at stages of III or IV of cancer (Table 1). It is well documented that hormone therapy [43, 44] and radiotherapy [45–48] have immunomodulatory effects. Toxoplasmosis is an opportunistic infection among patients with immunocompromising conditions [4]. Hence, the immunomodulatory effects induced by these treatments might increase the risk of toxoplasmosis.

In the current study, a negative association between *T. gondii* seropositivity with menopause status and *HER2* positivity were observed (Table 1). Although menopause leads to disturbance of the immune system, hormone therapy could revert this phenomenon [49, 50]. In our study, a significantly high seroprevalence rate of *T. gondii* was seen among *HER2*-negative patients compared with *HER2*-positive patients (74.57% vs. 10.41%, respectively, Table 1). It should be noted that *HER2*-positive patients have a better prognosis than *HER2*-negative patients [51]. Hence, this is an important point because toxoplasmosis could complicate the status of *HER2*-negative breast cancer patients. On the other hand, it is observed *T. gondii* induces epidermal growth factor receptor signaling in infected tissues to prevent parasite death by autophagy [52, 53]. As such, cerebral and retinal invasion by *T. gondii* are promoted through epidermal growth factor receptors [54]. Interestingly, suppressors of *HER2*/*4* have antitoxoplasmic activity *in vitro* [55].

Previous studies among cancer patients revealed that exposure to soil and consumption of raw/undercooked meat were among the risk factors of toxoplasmosis [56]. Additionally, other reports confirmed these risk factors as well as contact with cats, consumption of raw fruits and vegetables, and washing fruits and vegetables with untreated water [32, 33, 35, 36, 57, 58]. However, our study did not find a statistically significant association between *T. gondii* seropositivity and these factors (Table 1).

Lymphadenopathy is a common clinical manifestation of both breast cancer and toxoplasmosis [59]. In this regard, several cases of toxoplasmic lymphadenitis have been detected in axillary lymph nodes that mimic malignancy [21, 60–63].

Our results indicate that IgG-positive patients had previous exposure to the parasite and may have had a latent infection. It should be noted that reactivation of latent toxoplasmosis accounts for the majority of cases of central nervous system (CNS) toxoplasmosis during immunosuppression [8, 9, 11]. Hence, screening of toxoplasmosis could be recommended as a routine follow-up for immunocompromised patients, including patients with cancer [15, 64, 65].

### 4.1. Study Limitations and Suggestions for Future Researches

Our study has some limitations, including (1) limited number of samples, (2) we could not obtain the time of starting treatment for breast cancer, (3) PCR did not perform on biopsy specimens, and (3) we could not perform case-control study because our patients were from different cities in Fars Province, not only from Jahrom County. Hence, for preventing sampling bias, we performed a descriptive study among breast cancer who referred to our referral center to determine the demographic and associated risk factors of *T. gondii* infection among breast cancer patients. Based on these limitations, we should suggest that future studies should be perform on greater number of samples. Indeed, the time of starting treatment of cancer should be recorded to adjust for *T. gondii* infection. Furthermore, PCR will be performed on biopsy specimens alongside with blood samples. Additionally, we suggest case-control studies in future studies to compare the status *T. gondii* infection among breast cancer patients and healthy individuals.

## 5. Conclusion

Our findings showed a high seroprevalence rate of anti-*T. gondii* IgG in breast cancer patients. Although we did not detect active infection, reactivation of chronic infection due to immunosuppression should not be neglected among patients with malignancies. Nonetheless, such studies need to be done in a larger sample. Screening programs for detection of toxoplasmosis (due to reactivation of chronic infection) could be recommended as a routine follow-up among breast cancer patients.

## Figures and Tables

**Table 1:** Demographic characteristics, risk factors, and *T. gondii* infection status among 107 women with breast cancer.

Variables		Frequency (%)	Positive for anti-*T. gondii* IgG (%)	*P* value^∗^
Age (years)	34-51	68 (63.55)	36 (52.94)	0.22
	52-80	39 (36.44)	13 (33.33)	
Residence	Urban	95 (88.78)	45 (47.36)	0.55
	Rural	12 (11.21)	4 (33.33)	
Married	Yes	99 (92.52)	47 (47.47)	0.42
	No	8 (7.47)	2 (25)	
Education level	Illiterate	14 (13.08)	6 (42.85)	0.22
	Elementary and middle school	68 (63.55)	33 (48.52)	
	High school and above	25 (23.36)	10 (40)	
Number of children	0	3 (2.8)	1 (33.33)	0.08
	1	11 (10.28)	5 (45.45)	
	2	18 (16.82)	8 (44.44)	
	≥3	75 (70.09)	35 (46.66)	
Consumption of raw or undercooked meat	Yes	8 (7.47)	5 (62.5)	0.56
	No	99 (92.52)	44 (44.44)	
Washing raw fruits and vegetables	With disinfectant	17 (15.88)	9 (52.94)	0.69
	With water	90 (84.11)	40 (44.44)	
Type of water source	Treated pipe water	101 (94.39)	47 (46.53)	0.68
	Untreated (river, well, and rain water)	6 (5.6)	2 (33.33)	
Contact with soil	Yes	90 (84.11)	41 (45.55)	0.94
	No	17 (15.88)	8 (47.05)	
Contact with cats	Yes	17 (15.88)	7 (41.17)	0.79
	No	90 (84.11)	42 (46.66)	
Contact with domestic animals	Yes	23 (21.49)	11 (47.82)	0.89
	No	84 (78.5)	38 (45.23)	
Type of treatment	Surgery	15 (14.01)	4 (26.66)	0.08
	Radiotherapy	15 (14.01)	10 (66.66)	
	Hormone therapy (e.g., tamoxifen)	27 (25.23)	20 (74.07)	
	Unknown	50 (46.72)	15 (30)	
History of abortions	Yes	30 (28.03)	20 (66.66)	0.11
	No	77 (71.96)	29 (37.66)	
Time of abortion	First trimester	16 (53.33)	12 (75)	0.87
	Second trimester	8 (26.66)	5 (62.5)	
	Third trimester	6 (20)	3 (50)	
Menopause	Yes	48 (44.85)	8 (16.66)	**0.0005**
	No	59 (55.14)	41 (69.49)	
History of breast cancer	Yes	48 (44.85)	4 (8.33)	**0.0001**
	No	59 (55.14)	45 (76.27)	
*HER2* status	Positive	48 (44.85)	5 (10.41)	**0.00002**
	Negative	59 (55.14)	44 (74.57)	
Stage of cancer	I	8 (7.47)	2 (25)	0.51
	II	41 (39.75)	22 (53.65)	
	III	37 (34.57)	27 (72.97)	
	IV	21 (19.62)	15 (71.42)	

^∗^ *P* value <0.05 was considered significant. ^∗^Chi-squared test.

**Table 2:** Seroepidemiology of *Toxoplasma gondii* in women reported by previous studies from Fars Province, Iran.

Author	Year	Study city	Study population	Age (range or mean ± SD)	Sample size (n)	Infected (*n*, %)	Variables significantly associated with *Toxocara* infection
Davami et al. [31]	2013	Jahrom	Women who intend to marry	11–45 years	403	IgG (52, 13%), IgM (8, 2%)	No significant correlation
Maani et al. [25]	2022	Jahrom	Pregnant women who were in the first trimester of pregnancy	>20 ≤ 35 years	370	IgG (109, 29.5%), IgM (2, 0.5%)	A significant relationship between the frequency of IgG and the number of children (mothers with three children) was observed (*P* = 0.005)
Norouzi et al. [28]	2017	Shiraz	Pregnant women	15–45 years	2000	IgG (172, 8.6%), IgM (4, 0.2%), both IgG/IgM (1, 0.05%)	A significant relationship between the frequency of IgG and first trimesters of pregnancy was observed (*P* < 0.01)
Taghizadeh et al. [29]	2017	Shiraz	Female university students	22.2 ± 3.83 years	503	IgG (37, 7.4%), IgM (7, 1.4%), both IgG/IgM (1, 0.2%)	No significant correlation
Hatam et al. [30]	2005	Fasa	High school girls	14–19 years	947	IgG (96, 10%)	No significant correlation

## Data Availability

The data used in this study will be available upon authorization of the corresponding author.

## Abbreviations

*T. gondii*: *Toxoplasma gondii*
ELISA: Enzyme-linked immunosorbent assay
*HER2*: Human epidermal growth factor receptor 2
PCR: Polymerase chain reaction
*RE*: Repetitive element
CNS: Central nervous system.
